# Exercise interventions remodel the aging microenvironment to improve prognosis in older patients with cancer

**DOI:** 10.3389/fimmu.2026.1785073

**Published:** 2026-08-18

**Authors:** Wen Fang, Yihan Wang, Zhenyu Yang, Hongyun Zheng, Yang Wu, Ruogu Chen, Ruilong Wang, Huayu Tang, Menghao Tang, Xinming Ye

**Affiliations:** 1 School of Sports Science and Engineering, East China University of Science and Technology, Shanghai, China; 2 Animal Medicine, College of Life Sciences, Longyan University, Longyan, China; 3 School of Pharmacy, East China University of Science and Technology, Shanghai, China

**Keywords:** aging microenvironment, exercise intervention, geriatric cancer, immunosenescence, inflammaging, sarcopenia

## Abstract

Cancer in older adults has become a significant public health challenge, with increasing incidence and limited efficacy of conventional treatments due to aging-related biological changes, such as chronic inflammation, immune decline, metabolic dysfunction, and cellular senescence. These alterations create a pro-tumor environment, weakening antitumor capacity and reducing treatment sensitivity. Exercise medicine has emerged as a promising systemic intervention, regulating multiple physiological systems, including metabolism, immunity, skeletal muscle, and inflammation. Through these effects, exercise reshapes the aging-driven tumor-susceptible environment, potentially improving treatment tolerability and clinical outcomes. This review explores how exercise alleviates aging-associated phenotypes like metabolic decline, chronic inflammation, immune dysfunction, and sarcopenia, enhancing tumor suppression and boosting the efficacy of chemotherapy, radiotherapy, and immunotherapy. It also discusses strategies for precision exercise interventions, such as individualized prescriptions based on aging biomarkers, comprehensive risk assessments, safety management, multidisciplinary collaboration, and digital monitoring technologies. This review highlights the importance of integrating exercise into geriatric oncology care and outlines future research directions to improve the clinical management of cancer in older adults.

## Graphical Abstract

**Figure:**
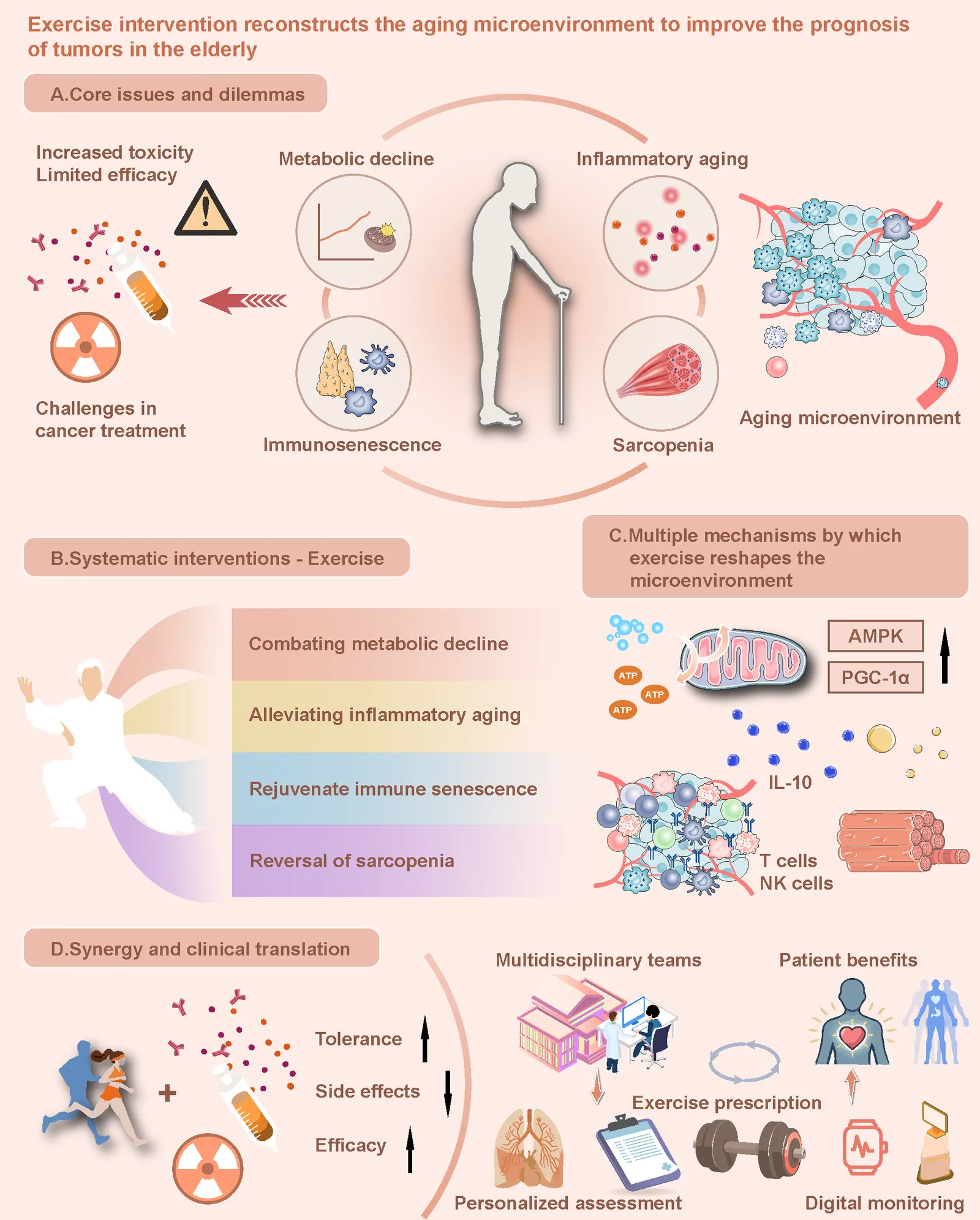
Exercise interventions help reshape the aging microenvironment to improve the prognosis of tumors in elderly cancer patients by addressing core aging-related challenges such as metabolic decline, inflammatory aging, immunosenescence, and sarcopenia. Regular physical activity combats metabolic dysfunction, alleviates chronic inflammation, rejuvenates immune function, and reverses sarcopenia, creating a more favorable internal environment for cancer treatment. Exercise enhances mitochondrial function, promotes anti-inflammatory mediators such as IL-10, and boosts the activity of T-cells and natural killer cells, improving immune surveillance and the tumor microenvironment. Furthermore, exercise intervention improves oxygen metabolism and blood supply to tumor tissues, reduces treatment-related side effects like radiation dermatitis and mucositis, and serves as a systemic toxicity buffer, ultimately synergizing with chemotherapy, radiotherapy, and immunotherapy to enhance treatment efficacy and patient outcomes.

## 1 Introduction

### 1.1 Rising burden of cancer in older adults and increasing treatment challenges

The global population is aging at an accelerating pace, accompanied by a marked increase in cancer incidence among older adults. Epidemiological data show that the number of newly diagnosed malignant tumors in individuals aged 60 years and above is growing steadily (1). For common cancers such as breast, colorectal, and prostate cancers, the median age at diagnosis has already surpassed 60 years (2, 3). The rising number of older patients with cancer is placing substantial pressure on public health systems and medical resources (4). Age related degenerative changes in organ function, along with the accumulation of chronic diseases, not only raise cancer risk but also contribute to increased complexity in tumor types among older individuals (5). The coexistence of multiple comorbidities and declining physiological reserves make these patients particularly vulnerable to complications during treatment. In this population, cancer often progresses more rapidly, is accompanied by more comorbid conditions, and presents with reduced treatment responsiveness (6). These factors create considerable challenges for conventional therapeutic approaches. For instance, older patients undergoing chemotherapy are more likely to experience severe bone marrow suppression and infections due to age associated decline in hepatic and renal function and reduced hematopoietic reserve (7). During radiotherapy, diminished tissue repair capacity contributes to more pronounced side effects (8). Surgical interventions are also associated with a significantly higher risk of postoperative complications in older patients (9).

Overlooking the frailty of older patients and administering standard treatment doses without adjustment can lead to severe adverse effects. Striking a balance between therapeutic efficacy and treatment safety remains a central challenge in geriatric oncology. Furthermore, older adults are frequently excluded from clinical trials, resulting in a lack of evidence-based guidelines tailored to this population. This gap in clinical evidence leaves physicians with limited support when making treatment decisions for older patients with cancer (10). There is an urgent need to develop integrated intervention strategies that not only effectively control tumor progression but also minimize treatment-related risks. As a safe, well-tolerated, and systemically beneficial approach, exercise has the potential to enhance the overall physiological function of older individuals (11). Its incorporation into cancer care may serve as a valuable adjunct to conventional therapies. Integrating exercise into the comprehensive treatment framework for older cancer patients may offer a novel avenue to address current therapeutic challenges.

### 1.2 Biological aging as a central driver of cancer risk and therapeutic failure

Aging is accompanied by a range of biological changes that increase the susceptibility of older individuals to cancer and introduce significant challenges to treatment (12). First, older adults commonly exhibit a state of chronic, low-grade inflammation, known as inflammaging, characterized by elevated levels of pro-inflammatory cytokines such as IL-6 and TNF-α (13). This persistent inflammation promotes oxidative stress and DNA damage, thereby increasing the risk of malignant transformation (14). It also activates tumor-promoting pathways such as NF-κB, which facilitates abnormal cell proliferation and angiogenesis, ultimately creating a favorable environment for tumor initiation and progression (15–18). Second, the decline in immune function associated with aging substantially weakens antitumor immunity (19). Thymic involution reduces the production of naïve T cells, NK cell activity is diminished, and the diversity of immune cell populations declines (20). These changes impair immune surveillance and the clearance of newly emerging tumor cells (21). As a result, the aging immune system becomes less capable of detecting and eliminating precancerous or cancerous cells, which not only increases cancer risk but also reduces the effectiveness of immunotherapy in older patients. Third, aging is associated with marked metabolic dysfunction, including insulin resistance, dysregulation of glucose and lipid metabolism, and mitochondrial impairment (22). These metabolic alterations compromise tissue repair capacity while simultaneously creating conditions that favor tumor growth. For instance, elevated levels of insulin and growth factors can stimulate tumor proliferation, while mitochondrial dysfunction and increased oxidative stress contribute to genomic instability and the accumulation of mutations (23). In addition, age-related increases in adipose tissue and reductions in skeletal muscle mass enhance the release of pro-inflammatory and pro-tumorigenic factors from visceral fat, further deteriorating the internal environment of the host (24).

Aging is also characterized by the accumulation of senescent cells that adopt a senescence-associated secretory phenotype (25). These cells release a variety of bioactive molecules, including interleukins, chemokines, and proteases (26). The presence of these factors contributes to chronic inflammation and promotes the degradation of the extracellular matrix (27). As a result, they can induce malignant transformation in neighboring cells and enhance tumor invasiveness and metastatic potential, ultimately diminishing the effectiveness of therapy (28).

The biological changes associated with aging increase cancer risk and reduce treatment efficacy at multiple levels (29). Tumor progression is not solely driven by the intrinsic properties of cancer cells but is also influenced by the aging status of the host (30). However, current therapeutic strategies tend to focus primarily on targeting tumor cells while overlooking host-related factors. This limitation often leads to suboptimal treatment outcomes in older patients, who may fail to respond adequately to standard therapies or may be unable to tolerate them, resulting in treatment discontinuation (31). Incorporating host aging characteristics into therapeutic planning represents a critical opportunity to address these deficiencies. A more comprehensive approach that integrates tumor biology with host aging physiology may improve treatment outcomes and provide more personalized and effective cancer care for older adults.

### 1.3 Exercise as a promising systemic intervention to overcome therapeutic barriers

In efforts to optimize cancer treatment strategies for older adults, exercise has gained considerable attention due to its systemic regulatory effects. Unlike traditional pharmacological therapies that often target specific molecular pathways, regular physical activity influences multiple physiological systems simultaneously and produces broad health benefits (32). One important effect of exercise is the improvement of energy metabolism (33). Moderate-intensity aerobic exercise enhances insulin sensitivity and reduces circulating levels of glucose and insulin, thereby addressing common disturbances in glucose and lipid metabolism seen in older adults (34). In addition, exercise promotes mitochondrial biogenesis, improves metabolic efficiency, and helps restore metabolic homeostasis (35). These changes provide essential energy support to counter the metabolic demands imposed by cancer and its treatment. Exercise also exerts anti-inflammatory and immunomodulatory effects that can help mitigate the decline in immune function associated with aging (36). By reducing visceral fat stores and promoting the release of anti-inflammatory mediators, exercise lowers levels of chronic inflammation (37). At the same time, it enhances immune function by increasing the activity of natural killer cells and effector T cells, promoting immune cell renewal, and facilitating the mobilization of immune cells into the circulation (38). These effects contribute to improved antitumor immunity (39). Furthermore, exercise plays a vital role in maintaining and improving muscle mass and physical capacity in older individuals (40). Age-related loss of muscle mass and strength, known as sarcopenia, reduces tolerance to disease and treatment stress (41). Targeted resistance and aerobic training can increase muscle strength and volume, enhance endurance and balance, and improve overall physical function (42). Greater muscle strength can help older patients better withstand the physiological demands of surgery and chemotherapy, and may reduce the risk of treatment-related complications (43).

Evidence indicates that appropriately screened and individually tailored exercise programs are feasible for many older adults with cancer, although implementation depends on comorbidity, functional status, treatment phase, access, and the availability of appropriate supervision (104, 205). In addition to its therapeutic potential, exercise improves cardiopulmonary function, enhances bone health, stabilizes mood, and contributes to better overall well-being (44). These improvements in quality of life can further strengthen patients’ confidence and ability to adhere to treatment regimens (45, 46). As a systemic intervention, exercise holds irreplaceable value in the management of cancer in older adults. By improving host physiology on multiple levels, exercise helps to create a more favorable internal environment for cancer therapy and may help overcome the persistent challenge of suboptimal treatment outcomes in this population. Although further evidence from clinical research and the development of innovative therapeutic models are still needed to guide implementation, current scientific understanding has already provided a solid foundation for the integration of exercise into comprehensive cancer care for older adults.

### 1.4 Objectives and key insights of this review

This review is developed in response to the increased vulnerability to cancer associated with aging and the potential of exercise to serve as a corrective intervention. It focuses on the concept that exercise may reshape tumor susceptibility linked to aging by acting across multiple biological levels to improve the overall health of older patients with cancer. The discussion begins with an examination of how aging-related biological changes influence tumor initiation, progression, and treatment response. It then explores how exercise can target physiological weaknesses commonly seen in older adults, with the goal of enhancing their antitumor capacity. The review proposes an integrated model that connects aging, exercise, and cancer across biological hierarchies. This model incorporates mechanisms from the molecular and cellular levels to entire organ systems, and spans from the tumor microenvironment to systemic physiological changes in the host. The central goal is to explain how exercise may improve resistance to cancer by modulating key vulnerabilities associated with aging. The review also evaluates the potential for exercise to act synergistically with conventional cancer therapies such as chemotherapy, radiotherapy, and immunotherapy, and considers the practical implications for clinical care. By synthesizing current scientific evidence and identifying future directions, this review aims to provide a theoretical foundation and practical guidance for applying exercise-based strategies in the treatment of cancer in older adults. This interdisciplinary perspective supports a shift in oncology from a tumor-centered approach to one that addresses both the tumor and the aging host, with the ultimate aim of improving outcomes for a larger population of older patients.

## 2 Exercise-mediated modulation of biological aging to enhance antitumor capacity

As aging progresses, the accumulation of biological alterations in older adults significantly weakens their intrinsic ability to resist tumor development and progression (47). Exercise, functioning as a systemic intervention, holds the potential to regulate aging-associated phenotypes and improve biological resilience (48). By targeting key mechanisms of aging, exercise may enhance the host’s capacity to combat cancer more effectively (49). This section explores how exercise strengthens antitumor capacity in older individuals by mitigating aging-related dysfunctions in several core systems. These include metabolic regulation, inflammatory control, immune function, and musculoskeletal integrity. Each of these domains plays a critical role in shaping the tumor microenvironment and the host’s physiological response to cancer, and they represent modifiable targets through which exercise can exert therapeutic benefit.

### 2.1 Metabolic decline and mitochondrial dysfunction

With advancing age, energy metabolism in older adults becomes progressively impaired (50). This decline is commonly manifested as a reduced basal metabolic rate, diminished metabolic flexibility, and a weakened ability to adapt to nutritional excess (51). Within the context of cancer, metabolic deterioration imposes a dual burden on older individuals. On one hand, insufficient energy supply contributes to treatment-related fatigue and unintended weight loss, both of which are prevalent among older patients undergoing cancer therapy (52). On the other hand, the aging metabolic environment may create favorable conditions for tumor growth (53). Dysregulation of glucose and lipid metabolism, together with insulin resistance, often leads to hyperglycemia and hyperinsulinemia (54). These metabolic disturbances provide an excess of energy and growth signals that can fuel tumor proliferation (55). In parallel, mitochondrial dysfunction compromises energy production and increases oxidative stress, which may initiate oncogenic pathways (56). The progressive loss of metabolic function not only diminishes the host’s physiological capacity to fight cancer but also facilitates tumor progression by creating an internal environment that supports malignant cell expansion (53).

**Figure 1:**
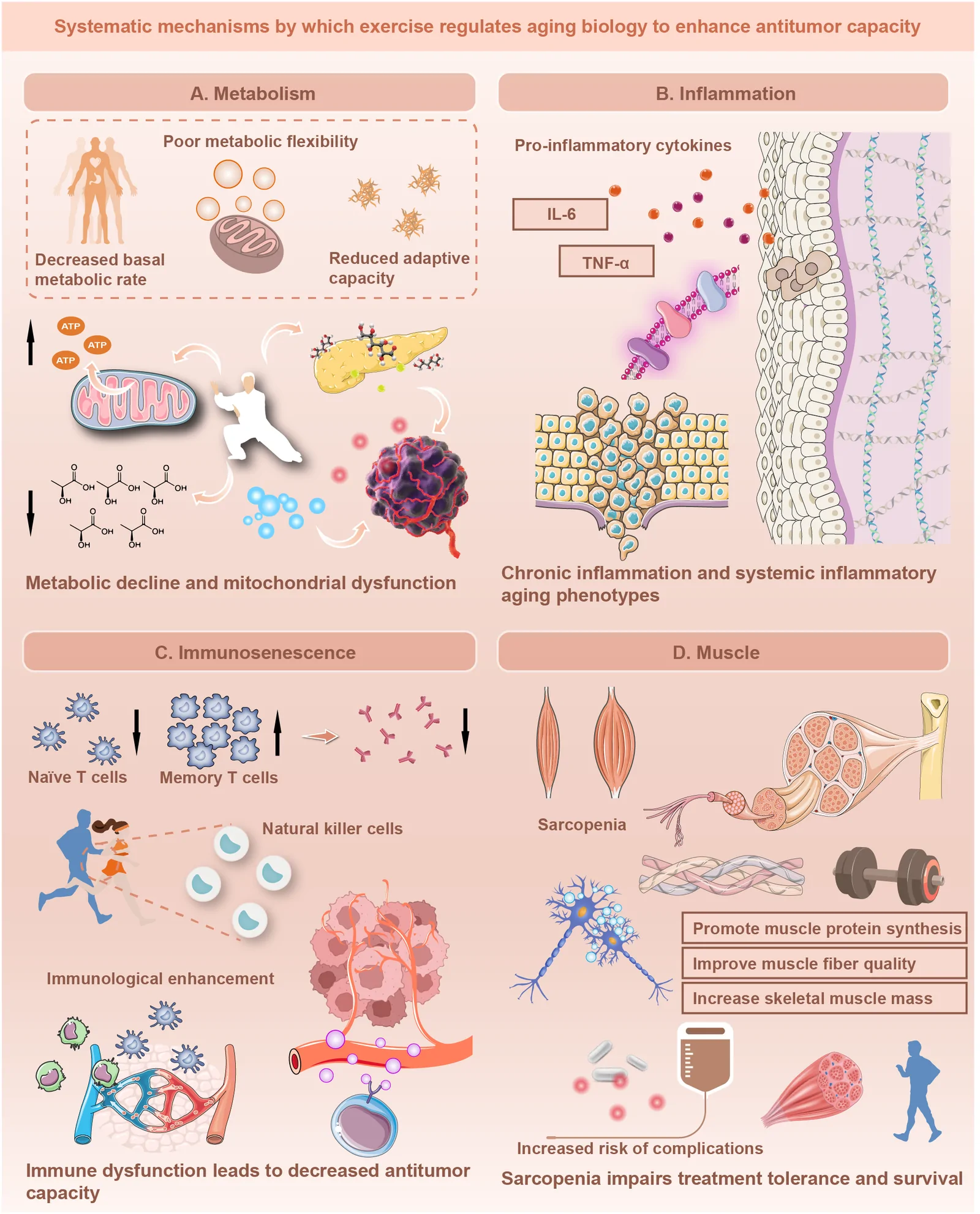
Systematic mechanisms by which exercise regulates aging biology to enhance antitumor capacity. **(A)** Metabolism: exercise helps improve poor metabolic flexibility and increases basal metabolic rate while enhancing adaptive capacity. These metabolic improvements counteract metabolic decline and mitochondrial dysfunction commonly associated with aging, providing better energy production and utilization, which is crucial for aging individuals and cancer patients undergoing treatment. **(B)** Inflammation: regular exercise reduces chronic inflammation and systemic inflammatory aging phenotypes by regulating pro-inflammatory cytokines like IL-6 and TNF-α. This modulation of inflammatory markers helps alleviate the aging-related inflammatory environment that supports tumor growth, metastasis, and resistance to treatments, promoting a healthier internal milieu for cancer therapy. **(C)** Immunosenescence: exercise enhances immunological functions by increasing the number of naïve T cells and memory T cells while boosting the activity of natural killer cells. This strengthens immune surveillance and helps prevent immune failure, thereby enhancing the body’s ability to fight tumors. Furthermore, exercise improves immune response efficiency and reduces the immunosuppressive conditions that often develop with aging and tumor progression. **(D)** Muscle: exercise promotes muscle protein synthesis, improves muscle fiber quality, and increases skeletal muscle mass, all of which help counteract sarcopenia. This improvement in muscle health not only boosts physical strength and endurance but also enhances treatment tolerance, reducing the risk of complications associated with cancer therapies. Stronger muscles also contribute to better overall recovery and viability during and after treatment.

Exercise training is widely recognized as an effective strategy for improving metabolic function. Regular aerobic activity promotes mitochondrial biogenesis and enhances mitochondrial performance, thereby increasing cellular energy production (57). At the molecular level, these effects are primarily mediated through the activation of AMP-activated protein kinase and the upregulation of peroxisome proliferator-activated receptor gamma coactivator 1-alpha (58). Endurance training is a strong stimulus for mitochondrial biogenesis and oxidative adaptation through AMPK–PGC-1α signaling, whereas resistance exercise more strongly stimulates myofibrillar protein synthesis and hypertrophic signaling through mTORC1; however, these responses overlap and are not mutually exclusive (244). These pathways contribute to an increase in mitochondrial content and oxidative phosphorylation capacity in skeletal muscle and other tissues (59). By restoring mitochondrial function through exercise, older adults can partially reverse the age-related decline in cellular energy generation, leading to improved energy availability for various tissues (60). In addition to enhancing mitochondrial efficiency, exercise also improves systemic metabolic flexibility (**Figure 1**) (61). This enables the body to switch more effectively between glucose and lipid utilization, thereby preventing the accumulation of metabolic byproducts and reducing the risk of metabolic imbalance (62). For older patients with cancer, these adaptations are particularly beneficial. Improved host metabolic resilience may help older patients better withstand cancer- and treatment-related fatigue, weight loss, and metabolic stress (63). Exercise may also alleviate treatment-related metabolic side effects and fatigue. Evidence suggests that moderate exercise can reduce chemotherapy-induced fatigue and improve appetite in cancer patients (64). These benefits are likely related to improved glucose utilization, enhanced clearance of lactate, correction of therapy-induced metabolic disruptions, and increased insulin sensitivity (65). Altogether, enhanced energy supply and restored metabolic homeostasis provide a stronger physiological foundation for older patients undergoing cancer treatment. This may translate into improved treatment tolerance and responsiveness.

### 2.2 Chronic inflammation and the systemic inflammaging phenotype

Chronic inflammation is a prominent biological hallmark of aging and is commonly referred to as inflammaging (66). In older adults, the immune system remains in a state of persistent low-grade activation, characterized by elevated levels of pro-inflammatory cytokines. This systemic inflammatory environment accelerates both the onset and progression of cancer (67). Persistent inflammation contributes to carcinogenesis by inducing genetic mutations and promoting genomic instability, thereby facilitating the transformation of normal cells into malignant ones (68). Key inflammatory mediators, such as IL-6 and TNF-α, activate downstream signaling pathways that support tumor cell proliferation and survival (69). Moreover, chronic inflammation recruits and polarizes immunosuppressive cells within the tumor microenvironment, fostering immune evasion by cancer cells. This process helps tumors escape immune detection and destruction (70). In addition, inflammation-induced tissue injury and fibrosis create structural and biochemical conditions that facilitate tumor invasion and metastasis (71).

The anti-inflammatory effects of exercise have been widely demonstrated. Regular physical activity can significantly reduce circulating levels of various pro-inflammatory cytokines (72). In older populations, moderate-intensity aerobic exercise has been shown to lower serum concentrations of inflammatory markers such as CRP and IL-6 (73). Chronic IL-6 elevation in inflammaging is associated with persistent pro-inflammatory signaling, whereas the transient rise in muscle-derived IL-6 during exercise forms part of an acute immunoregulatory response that may promote anti-inflammatory mediators such as IL-10; this functional distinction is context dependent rather than absolute (66, 73, 245, 246).

Exercise exerts its anti-inflammatory effects through multiple mechanisms (74). It helps reduce visceral fat accumulation, which is one of the primary sources of chronic inflammatory mediators. During physical activity, contracting skeletal muscles release anti-inflammatory myokines that counteract pro-inflammatory signaling (75). Beyond soluble cytokines, recent evidence highlights non-canonical communication channels, such as muscle-derived extracellular vesicles (EVs) containing exosomal miRNAs. These EVs act as critical mediators by which skeletal muscle structurally and immunologically remodels the distal tumor microenvironment, similar to the profound immunomodulatory roles miRNAs play in metabolic disorders (236). Exercise also improves the composition of the gut microbiota and enhances tissue perfusion, which in turn lowers the translocation of endotoxins and other inflammatory triggers into systemic circulation (**Figure 1**) (76). Specifically, exercise enriches microbial populations that produce short-chain fatty acids (SCFAs), most notably butyrate. This increase in butyrate strengthens the intestinal epithelial barrier and directly suppresses nuclear factor kappa B (NF-κB) signaling in local macrophages, thereby preventing the systemic translocation of lipopolysaccharide (LPS) endotoxins. The optimization of gut microbiota is increasingly recognized as a key regulator in preventing vascular inflammatory aging and extending systemic healthspan (237, 238). Through these pathways, exercise effectively alleviates the chronic low-grade inflammation commonly observed in older adults and helps restore immune homeostasis. Within the malignant cycle that links inflammation to tumor progression, exercise has the potential to disrupt this feedback loop. By reducing pro-tumorigenic inflammatory stimuli, exercise may suppress tumor cell proliferation, invasiveness, and metastatic potential (77, 78). In preclinical tumor models, exercise has been associated with lower intratumoral immunosuppressive cell abundance and greater cytotoxic immune-cell infiltration, changes that may support immune surveillance (86, 88). Therefore, exercise is not only a supportive approach to symptom control but may also attenuate inflammatory pathways that can promote tumor development (77, 78). In older patients who often carry a substantial inflammatory burden due to aging, the anti-inflammatory effects of exercise have meaningful clinical relevance. Exercise may serve as an adjunctive approach that combines anti-inflammatory and antitumor benefits in the management of cancer in the aging population.

### 2.3 Immunosenescence and the decline of antitumor immunity

The immune system plays a critical role in recognizing and eliminating tumor cells (79). However, immunosenescence, a hallmark of aging, is widely observed in older adults and significantly compromises their capacity to mount effective antitumor immune responses (80, 121). In aging individuals, T cell populations undergo marked alterations. The number of naïve T cells declines, while memory T cells become disproportionately expanded. This shift reduces the immune system’s ability to respond to novel tumor antigens (81). In addition, the functional capacity of effector T cells and NK cells is diminished, leading to reduced cytotoxicity and allowing tumor cells to evade immune surveillance more easily (82). At the same time, the proportion of regulatory T cells increases and myeloid-derived suppressor cells accumulate, contributing to a more immunosuppressive internal environment (83). These age-related immune changes are associated with impaired tumor immune surveillance and may contribute to tumor progression in older adults (82). Furthermore, such immunological alterations are associated with reduced responsiveness to immune checkpoint inhibitors, such as PD-1 or PD-L1 blockade therapies (84). In this context, relying solely on exogenous immunotherapeutic agents may be insufficient to fully reactivate the weakened immune systems of older patients. Effective cancer management in this population may require strategies that not only target the tumor but also aim to rejuvenate or support host immune function.

Encouragingly, exercise has been shown to act as a natural adjuvant that enhances immune function and holds significant potential in counteracting immunosenescence (19). Older adults who engage in regular moderate physical activity tend to have higher numbers and greater activity of NK cells in peripheral blood compared to their sedentary counterparts (85). This suggests that exercise can improve the efficiency of the innate immune system in tumor surveillance and clearance. Regular exercise also helps preserve the vitality of the T cell pool and enhances T cell responsiveness to antigens (39). Studies have demonstrated that physical activity can increase the number of immune cells and improve their functional capacity, thereby improving antitumor immune surveillance in the aging population (19). The beneficial modulation of the immune system by exercise may also translate into improved responses to immunotherapy. Exercise has been found to reduce the proportion of immunosuppressive cells, such as regulatory T cells and myeloid-derived suppressor cells, within the tumor microenvironment (86, 87). At the same time, it enhances the activity of infiltrating cytotoxic T lymphocytes and NK cells within tumors (88). These changes contribute to a more favorable microenvironment for immune checkpoint inhibitors and other immunotherapeutic strategies. In this context, exercise may help convert tumors from immunologically “cold” to “hot” states, thereby increasing treatment responsiveness in older patients (89). Additionally, exercise contributes to the improvement of systemic inflammatory and nutritional status, which may help mitigate adverse effects associated with immunotherapy and support treatment completion (90). Preliminary studies have suggested that exercise interventions may extend survival in animal models receiving immune-based treatments (91). As research in this area continues to advance, exercise is emerging as a promising and low-cost adjunct that could enhance the effectiveness of precision immunotherapy for older adults with cancer.

### 2.4 Sarcopenia reduces treatment tolerance and survival potential

Sarcopenia is highly prevalent among older adults with cancer and has been identified as a significant predictor of poor prognosis (92, 93). As an essential metabolic and reserve organ, skeletal muscle plays a critical role in maintaining physiological resilience (94). Severe muscle loss weakens a patient’s capacity to withstand both disease burden and treatment-related stress (95). In the context of cancer therapy, sarcopenia is associated with multiple adverse outcomes. Patients with reduced muscle mass are more susceptible to chemotherapy toxicity, in part due to a lower volume of drug distribution that results in higher effective drug concentrations (96). Pharmacokinetically, this is not merely an issue of a lower volume of drug distribution; low lean body mass fundamentally alters the metabolism and clearance of hydrophilic cytotoxic agents. Consequently, patients often experience higher effective drug concentrations and severe toxicities despite receiving standard body-surface-area (BSA)-based dosing. Inadequate protein reserves can impair postoperative tissue repair and increase the risk of infectious complications in the perioperative setting (97). Individuals with sarcopenia are more likely to experience treatment interruptions or dose reductions during surgery, radiotherapy, or systemic therapy, which can significantly elevate the risk of tumor recurrence and mortality (98). For these reasons, muscle condition has become an important indicator of frailty in older patients with cancer. However, a clear clinical distinction must be made between primary age-related sarcopenia, which is highly responsive to mechanical loading and resistance training, and tumor-induced cancer cachexia. Cancer cachexia represents a profound hypercatabolic state driven by systemic tumor-derived inflammatory factors (such as TNF-α and proteolysis-inducing factor) where mechanical loading alone is insufficient and requires multi-modal metabolic and nutritional support. Progressive resistance exercise is a core non-pharmacological component of sarcopenia management and is ideally combined with individualized nutritional support (200, 209). Properly designed resistance training programs can effectively stimulate muscle protein synthesis, improve muscle fiber quality, and increase skeletal muscle mass (99). Even in very old patients, resistance training tailored to individual capabilities has been shown to produce meaningful improvements in muscle volume (100). When combined with aerobic exercise, these interventions also enhance muscular endurance and cardiopulmonary function, improving the efficiency of oxygen and nutrient utilization within muscle tissue (101). Exercise training further improves neuromuscular coordination, increases muscle strength, enhances motor control, and reduces the risk of falls and mobility impairments (102, 103). Through systematic exercise interventions, older patients can expand their physiological reserves and become better prepared to complete intensive cancer treatment cycles (**Figure 1**) (104). Adequate muscle mass supports improved tolerance to therapy and facilitates adherence to treatment plans, which in turn contributes to better therapeutic outcomes (105). Studies have shown that improving muscle health through exercise can significantly reduce chemotherapy discontinuation rates and the incidence of severe adverse effects (106). Enhanced muscle strength also improves functional independence, reduces fatigue, and enhances quality of life, all of which help sustain patients’ confidence in continuing treatment (107). Over the long term, these benefits may contribute to improved survival. Therefore, maintaining and enhancing muscle health through exercise can help older patients with cancer achieve better outcomes throughout the treatment process.

## 3 Multilevel mechanisms through which exercise remodels the aged microenvironment to suppress tumor progression

To understand how systemic alterations drive the local remodeling of the tumor microenvironment (TME), it is essential to delineate the systemic-to-local delivery mechanisms.

### 3.1 Characteristics of the aged microenvironment and its role in promoting tumor progression

Building upon the systemic host factors associated with aging discussed in Section 2 (e.g., systemic inflammaging and metabolic decline), this section focuses on how these changes are associated with local remodeling of the tumor microenvironment (TME). These systemic physiological changes are linked to local features of the aged TME that can favor tumor progression. Tumor initiation and progression are influenced not only by intrinsic cancer-cell properties but also by the surrounding tissue microenvironment (108, 109). In older individuals, the tissue microenvironment undergoes age-related remodeling and exhibits several features that actively support tumor development and progression (110). One key feature is the accumulation of senescent cells and their release of senescence-associated secretory phenotype factors. These secreted molecules can induce malignant transformation in neighboring epithelial cells, enhance tumor cell survival, and recruit immunosuppressive cells (111). Together, these effects promote tumor invasion and increase resistance to therapy (112). Another hallmark of the aged microenvironment is the stiffening and fibrosis of the extracellular matrix (113). A hardened matrix facilitates tumor cell invasion and migration by providing pathways for dissemination and allowing cancer cells to breach tissue barriers (114). Excessive matrix rigidity also promotes tumor proliferation and resistance to apoptosis through mechanotransduction pathways (115). At the same time, structural abnormalities in the extracellular matrix may hinder the infiltration of immune cells, thereby impairing the antitumor immune response (116). Vascular degradation is another characteristic of the aged microenvironment (117). Capillary rarefaction and endothelial dysfunction reduce tissue perfusion in older individuals, resulting in a hypoxic and nutrient-deprived tumor microenvironment (118). In addition, lactate accumulation and extracellular acidification can suppress anticancer immunity and promote tumor aggressiveness (134). Hypoxia promotes tumor aggressiveness by activating pathways such as hypoxia-inducible factor 1 alpha and also enhances resistance to chemotherapy and radiotherapy (119). Furthermore, vascular deterioration limits the delivery of anticancer drugs and immune cells, further compromising therapeutic efficacy (120). The immune landscape of the aged microenvironment is also skewed toward tumor tolerance. Age-related immune dysfunction includes impaired T cell activity and increased accumulation of immunosuppressive cell populations (121). Together, these factors create a permissive environment for tumor immune evasion. Through these converging mechanisms, the aged microenvironment becomes an active enabler of cancer progression, providing multifaceted support for tumor growth, invasion, and resistance to treatment.

Traditional cancer treatments primarily focus on eliminating tumor cells while often neglecting the role of the surrounding host tissue microenvironment. This oversight can limit therapeutic effectiveness. Even when a portion of cancer cells is successfully eradicated, residual malignant cells may rapidly repopulate by exploiting a favorable microenvironment (122). In recent years, oncology has increasingly emphasized therapeutic strategies that target the tumor microenvironment (123). These include approaches such as eliminating senescent cells, suppressing the senescence-associated secretory phenotype, and remodeling abnormal extracellular matrix structures (124). Such strategies aim to disrupt the supportive conditions that tumors rely on for growth and survival. Exercise, as a systemic intervention, has demonstrated potential in optimizing the aged microenvironment. By modifying key features of tissue aging, exercise may indirectly hinder tumor progression. This has led to growing interest in exercise as a promising adjunctive approach in cancer management, particularly in older populations.

### 3.2 Exercise enhances tumor tissue oxygen metabolism and blood supply

**Figure 2:**
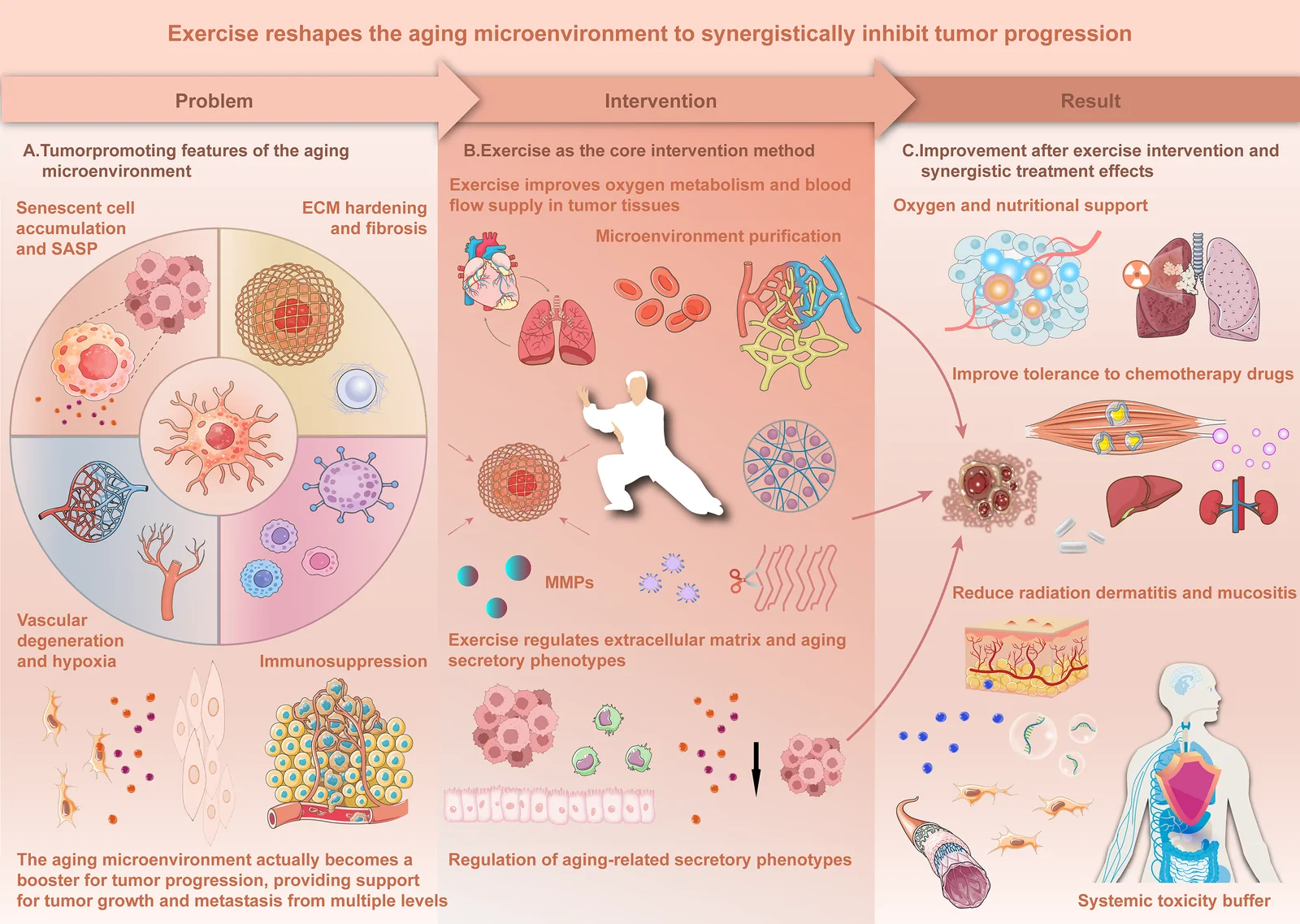
Exercise reshapes the aging microenvironment to synergistically inhibit tumor progression. **(A)** Tumor-promoting features of the aging microenvironment: The aging microenvironment, characterized by senescent cell accumulation, ECM hardening, vascular degeneration, and immunosuppression, actively supports tumor growth and metastasis at multiple levels, promoting an environment conducive to cancer progression. **(B)** Exercise as the core intervention method: Exercise improves oxygen metabolism, enhances blood flow supply to tumor tissues, and regulates the extracellular matrix and aging-related secretory phenotypes, contributing to microenvironment purification and improving conditions for cancer treatment. **(C)** Improvement after exercise intervention and synergistic treatment effects: Exercise intervention improves oxygen and nutritional support, boosts tolerance to chemotherapy, reduces radiation dermatitis and mucositis, and serves as a systemic toxicity buffer, enhancing the effectiveness of cancer treatments.

Exercise improves tumor tissue oxygenation and perfusion by enhancing cardiopulmonary and circulatory function (**Figure 2**) (125). In a breast cancer model, a 2-week period of daily exercise training increased tumor perfusive capacity and was associated with greater CD8+ T-cell infiltration (127). Studies have shown that aerobic training can promote vascular remodeling in tumors in certain animal models. This includes the formation of new microvessels and improved microcirculatory perfusion (128). However, it is crucial to distinguish this from pathological angiogenesis; the therapeutic goal is not to indiscriminately promote pro-tumorigenic angiogenesis or simply increase vessel density. Instead, exercise acts through the modulation of the HIF-1α/VEGF axis to reduce vessel tortuosity and leakiness, thereby contributing to the vascular normalization of tumor vasculature (129). Consistent with these preclinical findings, a secondary transcriptomic analysis of tumors from a preoperative exercise RCT in breast cancer found a greater reduction in a prognostic hypoxia gene signature in the exercise group; the overall comparison was marginal, with a stronger signal in grade 3 tumors (126). It enhances the maturity and structural integrity of blood vessels, making them functionally more similar to those found in healthy tissue (130). From a clinical translation perspective, this structural normalization induces a temporary, acute “window of opportunity” lasting several hours post-exercise, during which tumor tissue perfusion peaks and microenvironmental hypoxia is lowest, marking the ideal clinical timeframe for optimizing the delivery and cytotoxic impact of infused chemotherapeutic agents. Such changes improve oxygen gradients within the tumor, reduce the presence of severely hypoxic zones, and suppress the proliferation and genetic instability of cancer cells (127). As a result, tumors become less aggressive and more responsive to treatment.

As tumor tissue perfusion and oxygen availability improve, treatment efficacy is expected to be enhanced. Adequate perfusion facilitates more efficient delivery of chemotherapeutic agents into the tumor core, increasing drug exposure for cancer cells (131). Radiotherapy also relies heavily on the presence of oxygen, as oxygen enhances the formation of free radicals induced by radiation, thereby increasing damage to tumor cells (132). By alleviating tumor hypoxia, exercise has the potential to improve the effectiveness of radiotherapy. In addition to enhancing the performance of current treatments, exercise may also reduce the risk of treatment resistance. Many resistance mechanisms are linked to local hypoxia and uneven drug distribution within tumors (136). Exercise improves tissue perfusion and oxygenation, exposing tumor cells to a more consistent and therapeutically favorable environment. This can make cancer cells more susceptible to immune recognition and therapeutic targeting (130). These observations support continued investigation of exercise as a modulator of tumor biology (125, 130, 135). In summary, exercise supports the suppression of tumor progression by optimizing oxygen delivery and metabolic conditions within tumor tissue. These improvements help overcome barriers to effective treatment and contribute to better clinical outcomes (137).

### 3.3 Exercise regulates the extracellular matrix and senescence-associated secretory phenotype

The extracellular matrix and senescence-associated secretory factors within the aged microenvironment play critical roles in promoting tumor progression (138). Exercise modulates these components through multiple mechanisms and may help restore a healthier tissue milieu that suppresses cancer development (**Figure 2**). One important mechanism is the mechanical stimulation induced by physical activity, which promotes the reorganization and renewal of collagen fibers in tissues (139, 140). This process helps prevent excessive collagen cross-linking and the resultant stiffening of the matrix. Exercise also stimulates muscle and fibroblast cells to release matrix metalloproteinases, which contribute to the degradation of accumulated abnormal extracellular components (141). Exercise-associated mechanical loading and tissue remodeling may influence extracellular-matrix turnover, although direct tumor-specific mechanisms remain incompletely defined (139–142). A healthier matrix structure also enhances the migration of immune cells by providing a more accessible pathway through the tissue, thereby strengthening immune responses within the tumor microenvironment (142).

A further important effect of exercise is its ability to regulate the senescence-associated secretory phenotype. Senescent cells release a complex array of factors that contribute to inflammation and tumor promotion (28). By reducing the accumulation of senescent cells and suppressing inflammatory responses, exercise helps to lower the levels of these secretory factors in the tissue environment (143). Evidence from research indicates that long-term physical activity significantly reduces the expression of cellular senescence markers, suggesting a decrease in the number of senescent cells (144). In addition, exercise enhances immune surveillance, enabling the immune system to better identify and eliminate senescent cells. This contributes to a reduction in the production of pro-tumorigenic and pro-inflammatory secretory signals at their source (145). Taken together, exercise effectively counteracts the tumor-promoting influence of the senescence-associated secretory phenotype by improving the inflammatory balance and strengthening immune function within the aged microenvironment. These changes help to slow the progression of cancer.

## 4 Biological mechanisms underlying the synergistic effects of exercise on treatment responsiveness in older adults with cancer

### 4.1 Exercise improves tolerance and efficacy of chemotherapy and radiotherapy

The ability of cancer patients to complete chemotherapy and radiotherapy often depends on their physical fitness and the functional reserve of vital organs (146). Older adults, who frequently experience diminished physiological capacity, are more likely to struggle with treatment-related toxicity (147). Exercise, through its systemic regulatory effects, significantly improves the overall physiological condition of older patients and serves as a protective mechanism during cancer treatment (148). One benefit of exercise is the enhancement of cardiopulmonary function. By improving aerobic capacity and circulatory efficiency, exercise helps ensure adequate oxygen and nutrient delivery during therapy (149). This support can reduce damage to normal tissues, alleviate treatment-related fatigue, and lower the risk of radiation-induced lung injury and cardiovascular complications (104, 150, 240).Another important benefit is the improvement of muscular and hepatic-renal metabolic function. Exercise increases hepatic blood flow and enhances enzymatic activity, which promotes more efficient drug metabolism and clearance (151–154). This helps reduce the accumulation and toxicity of chemotherapeutic agents. In addition, increased muscle mass improves the distribution of medications throughout the body, minimizing the risk of high localized drug concentrations that could damage specific tissues (155).

By improving the internal environment and supporting organ function, exercise greatly reduces the risk of severe adverse effects in older patients. Many older individuals are forced to postpone or discontinue chemotherapy due to bone marrow suppression or generalized frailty (156). Exercise helps maintain bone marrow activity and supports immune function, thereby lowering the incidence of severe neutropenia and infections (157). This contributes to uninterrupted treatment delivery and improves therapeutic consistency. During radiotherapy, patients who engage in moderate exercise are less likely to experience serious side effects such as radiation-induced dermatitis and mucositis (158). Improved treatment tolerance allows patients to complete therapy according to schedule, ensuring the intended therapeutic dose is fully administered (159). In addition, exercise contributes to improved treatment outcomes by promoting tissue repair and regeneration (160). Chemotherapy and radiotherapy inevitably cause damage to healthy tissues. Exercise facilitates recovery by enhancing local blood flow, mobilizing stem cells, and stimulating the secretion of growth factors. For instance, aerobic exercise can mobilize bone marrow progenitor cells and support the recovery of blood cells (161). The shear stress generated during physical activity also promotes endothelial repair and reduces radiation-induced vascular damage (162, 163). Accelerated healing of normal tissues enables patients to proceed more quickly to the next cycle of treatment, reducing delays and minimizing the cumulative effects of tissue injury. This exercise-induced enhancement in tissue repair and metabolic glucose regulation is highly consistent with rehabilitation outcomes observed in other severe tissue trauma models (241, 242). Overall, exercise optimizes the internal physiological environment and strengthens the capacity of older patients to respond to and tolerate cancer therapy.

### 4.2 Potential mechanisms through which exercise enhances the effectiveness of immunotherapy

Immunotherapy has marked a major advancement in cancer treatment. However, older patients often experience diminished efficacy due to age-related immune dysfunction (164). Exercise has the potential to partially bridge this gap and improve immune responsiveness in aging individuals. One important mechanism is the increased infiltration and activation of immune cells within the tumor microenvironment. Preclinical and translational evidence suggests that exercise can mobilize and enhance the activity of effector T cells and natural killer cells (19, 39, 127). These immune cells play a central role in mediating the effects of treatments such as immune checkpoint inhibitors (165, 166). Experimental studies have demonstrated that tumors in physically active mice exhibit higher densities of CD8-positive T lymphocytes compared to sedentary controls, indicating a more robust antitumor immune response (127). In older patients, exercise may help shift tumors from an immunologically inactive state to one that is more immunologically responsive. This transformation could enhance the effectiveness of therapies such as PD-1 and PD-L1 inhibitors or chimeric antigen receptor T cell treatments (19).

Another key mechanism involves the ability of exercise to reverse or mitigate immunosuppressive factors within the tumor microenvironment, thereby improving the efficacy of immunotherapy (165). Following exercise intervention, levels of immunosuppressive cells and signaling molecules within tumors, such as regulatory T cells, myeloid-derived suppressor cells, and TGF-β, are often reduced (167, 168). Preclinical studies indicate this immunosuppressive reversion is driven by specific chemokine alterations, notably the upregulation of CXCR3 ligands (CXCL9/CXCL10) alongside the downregulation of Treg-recruiting CCL22, alongside an exercise-induced drop in local hypoxia that deactivates the HIF-1α signaling cascades required for myeloid-derived suppressor cell (MDSC) survival. This reduction enhances the activity of immune checkpoint inhibitors and allows them to function more effectively (86). Exercise also lowers systemic chronic inflammation, which can help reduce some of the adverse effects associated with immunotherapy (169). A lower baseline level of inflammation decreases the risk of excessive immune responses during treatment, improving its safety profile (170). In addition, studies have shown that exercise reduces immunotherapy-related fatigue and muscle wasting, helping patients maintain better physical condition throughout treatment (49). Over the long term, exercise may also enhance the durability of clinical responses to immunotherapy (38). One of the intended outcomes of immune-based cancer treatment is the development of long-lasting immune memory that can surveil and prevent tumor recurrence (171). Exercise supports this objective by improving immune system function and potentially strengthening the formation of memory immune cells (172). Research has indicated that exercise enhances the function of secondary lymphoid organs, including lymph nodes and the spleen, which contributes to the generation of high-quality and persistent memory T cell populations (173). Furthermore, the improved metabolic environment associated with regular physical activity provides a more favorable niche for the survival and maintenance of memory cells (174, 175). Although this area of research is still in its early stages, integrating exercise into immunotherapeutic regimens holds promise for extending progression-free and overall survival in older patients with cancer. In summary, exercise enhances the effectiveness of immunotherapy by improving the function of effector immune cells, reducing immunosuppressive signals, and promoting the formation of durable immune memory.

### 4.3 Exercise as a buffer against multisystem toxicities of cancer treatment

Cancer treatment is frequently accompanied by a variety of systemic side effects, affecting both physical and psychological aspects of health (176). Exercise serves as an effective buffer by positively regulating multiple physiological systems, thereby helping to alleviate these adverse effects. Cancer-related fatigue is a common issue faced by many patients, particularly among older individuals (177). Exercise is considered one of the most effective interventions for reducing fatigue. Although it requires energy expenditure, moderate physical activity can gradually improve functional capacity and exercise tolerance, which may lessen perceived fatigue (178). Exercise also promotes the release of endorphins and improves sleep quality, both of which help enhance mental vitality (179). Studies have shown that patients participating in exercise programs report significantly lower levels of fatigue compared to control groups, both during chemotherapy and in the post-treatment recovery phase (180). Exercise also protects systems such as the cardiovascular and musculoskeletal systems, helping to reduce the damage caused by treatment. For example, some chemotherapeutic agents are associated with cardiotoxicity, while regular aerobic exercise can strengthen cardiac function and improve vascular endothelial health, thereby lowering the risk of cardiac dysfunction (181). In cases of radiotherapy-induced lung injury, respiratory training and aerobic activity can promote compensatory function and reconstruction of lung tissues (182). Exercise may also assist symptom management in breast cancer–related lymphedema (133). In addition, exercise improves appetite and enhances insulin sensitivity, helping to optimize nutritional metabolism (183). Many older patients experience appetite loss and weight reduction during treatment (184). Exercise stimulates gastrointestinal motility and digestive fluid secretion, which can help alleviate appetite issues (185). Increased muscle activity also boosts metabolic efficiency, reducing the likelihood of metabolic disorders such as elevated blood glucose levels (186).

The positive effects of exercise on psychological well-being and cognitive function are equally important. Older cancer patients often experience psychological issues such as anxiety and depression due to disease burden and treatment-related stress (187). Moderate physical activity has been shown to effectively alleviate these symptoms. Neurotransmitters such as dopamine and serotonin, which are released during exercise, contribute to emotional stabilization (188). The neuroprotective benefits of exercise are particularly relevant for older patients. Cancer itself, along with certain chemotherapeutic agents, can lead to cognitive decline, commonly referred to as chemo brain (189). Regular physical activity, especially exercises that incorporate coordination and balance training, can enhance cerebral blood flow and the expression of neurotrophic factors, which help mitigate cognitive deterioration (190–192). Preclinical studies link exercise-induced PGC-1α/FNDC5 signaling to hippocampal BDNF expression and neuroprotective pathways; its relevance to chemotherapy-related cognitive impairment in older patients remains to be established (247). Furthermore, emerging single-cell evidence demonstrates that exercise directly impacts neuroinflammatory responses by regulating specific microglial subtypes, offering profound neuroprotection (239). Improvements in psychological and cognitive function not only enhance the overall quality of life but also increase patients’ confidence in and adherence to treatment (193).

## 5 Precision pathways and implementation strategies for exercise interventions in older patients with cancer

### 5.1 Stratified exercise prescription based on aging biomarkers

The population of older cancer patients is highly heterogeneous, with significant variation in biological aging status and functional reserves (12). Therefore, when designing individualized exercise interventions, it is essential to incorporate aging-related biomarkers and phenotypic characteristics. Assessing inflammatory and immune markers can provide insight into the patient’s chronic inflammatory burden and immune status. For instance, serum CRP and IL-6 levels can reflect the degree of inflammation, while lymphocyte subset analysis can help evaluate the extent of immunosenescence (194). Furthermore, advanced biological aging assessments can incorporate cellular senescence biomarkers, such as monitoring p16INK4a gene expression levels in peripheral blood T-cells, providing clinicians with a sensitive molecular metric to track host biological age and evaluate long-term molecular responses to targeted physical activity. For patients with elevated inflammatory markers and impaired immune function, exercise prescriptions should focus on anti-inflammatory effects and immune enhancement. In such cases, moderate-intensity aerobic exercise is recommended, with gradual adjustments in volume based on individual tolerance (195).

In addition, exercise planning can be guided by metabolic indicators and body composition measures (196). Metabolic health markers such as fasting blood glucose and lipid levels, together with body composition analysis including measurements of muscle and fat mass, help to assess a patient’s metabolic status and the risk of sarcopenia or obesity (197, 198). For patients with metabolic abnormalities, such as elevated blood glucose, and insufficient muscle mass, exercise prescriptions should focus on improving insulin sensitivity and include resistance training to increase muscle mass (199). For patients with low body weight and significant muscle loss, the emphasis should be on resistance training along with enhanced nutritional support (200). Exercise prescriptions guided by these biological markers can be adjusted in a personalized manner in terms of intensity and frequency. For example, physiological indicators such as heart rate, blood pressure, and subjective fatigue can be monitored to adjust exercise intensity dynamically, ensuring that the training is effective while remaining within safe limits (201, 202). Resistance training loads can be selected according to results from muscle strength tests, such as using specific percentages of one-repetition maximum, and the frequency and recovery periods can be adjusted based on training outcomes (203). Regular monitoring of key biological indicators, such as changes in inflammatory markers and increases in muscle mass, allows effective assessment of the impact of the exercise intervention and timely optimization of the exercise prescription (204). Crucially, these biomarker-guided prescriptions must avoid a one-size-fits-all approach and be explicitly tailored to distinct clinical windows. For example, the mechanistic goal of preoperative prehabilitation is the rapid expansion of physiological reserve to withstand acute surgical stress, whereas the goal for a survivor in remission focuses on long-term functional restoration, metabolic homeostasis, and recurrence prevention. These vastly different biological objectives necessitate phase-specific dosing and modality strategies. Through this precision stratification approach to management, exercise interventions can be better integrated into individualized treatment protocols for older patients with cancer, providing tailored support for each individual.

### 5.2 Individualized exercise design based on clinical risk and safety considerations

Safety must always be the foremost concern when implementing exercise interventions for older patients with cancer (205). Therefore, a comprehensive risk assessment is essential before designing an exercise program to ensure that the prescribed activities are both effective and free from harm. To establish absolute safety boundaries, practitioners must cross-reference clinical thresholds for absolute contraindications; exercise testing and training must be completely withheld in patients exhibiting severe chemotherapy-induced thrombocytopenia (platelets <25,000/μL), highly unstable bone metastases with imminent fracture risk, severe symptomatic anemia (hemoglobin <8 g/dL), or acute systemic infection accompanied by active fever. Patients should be evaluated for their risk of falls and musculoskeletal injuries due to factors such as impaired balance, osteoporosis, or degenerative joint conditions (206). For patients at higher risk, exercise forms with greater safety should be prioritized. Examples include seated exercises or water-based aerobic activities, which reduce joint strain and the risk of falls (207). In the early stages, assistive devices or professional supervision may be provided until balance and strength show improvement (208). Assessment of the risk of cardiopulmonary events is equally important. Older adults often have comorbid cardiovascular conditions and may experience symptoms such as chest discomfort or arrhythmias during exercise (209). Patients with a history of heart disease or other risk factors should undergo cardiopulmonary evaluation before beginning an exercise regimen, such as an exercise electrocardiogram or cardiopulmonary exercise testing (210). The results of these assessments should be used to establish safe limits for exercise intensity. During training sessions, vital signs including heart rate and blood pressure should be closely monitored. If symptoms such as chest tightness or dizziness occur, exercise should be stopped immediately (211). For patients with limited cardiac function, intermittent exercise patterns that alternate brief periods of activity with rest may help reduce the burden on the heart (212).

Cognitive and psychological factors must also be considered in exercise program design. For patients with cognitive impairments, exercise routines should be simplified and conducted under the supervision of family members to ensure safety (213). For those experiencing depression or anxiety, psychological counseling and motivational strategies should be used to help them understand the benefits of exercise, thereby enhancing their confidence and adherence (214). At the beginning of the intervention, exercise goals should be modest and achievable to prevent frustration (215). During the implementation phase, the program should be dynamically adjusted based on the patient’s changing condition. Physical capacity in older cancer patients may fluctuate throughout the course of treatment. For instance, exercise intensity may need to be reduced during periods of fatigue following chemotherapy, while it can be gradually increased during recovery phases (216). Therefore, a regular follow-up and assessment system should be established to monitor patients’ exercise tolerance and physical indicators. Based on this information, exercise prescriptions can be refined to better suit individual needs. The establishment of a safety monitoring system is also crucial. This includes equipping exercise settings with emergency supplies and medications, and ensuring continuous supervision by trainers or medical personnel to address any acute incidents (217). In conclusion, only by carefully balancing risks and benefits and tailoring programs to individual needs can safety risks be minimized and the benefits of exercise for older patients be fully realized.

### 5.3 Digital exercise monitoring and remote health management technologies

Advances in technology have created new opportunities for exercise management in older patients with cancer. Digital monitoring and remote health management technologies can significantly enhance the precision, reach, and adherence of exercise interventions (218). Wearable devices are increasingly used to monitor physical activity. Devices such as smart wristbands and heart rate chest straps can provide real-time tracking of heart rate, step count, and energy expenditure (219, 220). More advanced sensors are capable of measuring gait patterns, balance, muscle electrical activity, and blood oxygen saturation (221). These objective data provide healthcare professionals with precise information about exercise intensity and physiological responses. For example, analysis of heart rate data can help determine whether a patient has reached the target training heart rate range (222). Step count records offer feedback on the patient’s daily activity levels (223). With this data supported monitoring, both the adherence to exercise prescriptions and the evaluation of intervention outcomes become more quantitative and evidence based.

Heart rate variability can provide non-invasive information on autonomic regulation and recovery status, whereas inflammatory biomarkers such as IL-6 and CRP should be measured directly when clinically relevant (202, 204). Remote exercise prescription and realtime feedback systems can also significantly improve patient adherence (224). For patients with limited mobility or those living far from healthcare facilities, internet-based platforms allow for remote guidance. Physicians and rehabilitation specialists can use video conferencing to demonstrate movements, correct form, and adjust exercise intensity based on patient feedback in real time. Patients can receive professional guidance without leaving home (225). Supporting mobile applications can remind patients of their daily exercise plans and record progress through completion logs. The system can provide positive feedback automatically, which helps motivate active participation (226). By analyzing data from wearable devices and applications, the healthcare team can identify issues with exercise adherence in a timely manner and take appropriate action. For example, if the system detects a decline in a patient’s activity levels, it can alert medical staff to initiate follow-up and adjust the exercise plan as needed (227).

Building an intelligent health platform that integrates the above functions can further promote the large-scale implementation of exercise interventions. An ideal platform would connect patients, physicians, rehabilitation therapists, and family members to form a closed-loop management system. Physicians would formulate overall strategies, therapists would provide specific guidance, patients would upload their data, and the system would conduct intelligent analysis and provide feedback to the healthcare team. Such a system could not only offer personalized exercise interventions for each patient but also support future research and intervention optimization through the accumulation of real-world data. By leveraging digital and remote technologies, older adults can receive continuous exercise guidance and monitoring even in areas with limited medical resources. Ultimately, this would help achieve the goal of making rehabilitative exercise accessible to everyone.

### 5.4 Practice pathways for multidisciplinary collaboration in older patients with cancer

In order to effectively integrate exercise interventions into the treatment of older cancer patients, multidisciplinary collaboration is essential. The clinical complexity of older patients with cancer often exceeds the capacity of a single department to meet all of their needs, and the implementation of exercise interventions requires coordinated efforts from professionals across multiple fields (228). A multidisciplinary team should be established, including oncologists, exercise rehabilitation specialists, dietitians, psychologists, nurses, and social workers. Oncologists are responsible for developing treatment plans and ensuring patient safety. Exercise specialists design and guide individualized exercise programs. Dietitians ensure that nutritional needs are met. Psychologists support emotional well-being and help strengthen patient confidence. Nurses and social workers provide follow-up support and assistance with home care. Through multidisciplinary collaboration, patients can benefit from comprehensive support throughout their treatment journey, drawing on the expertise of professionals from different disciplines.

The advantage of multidisciplinary collaboration lies in its ability to enhance the quality of intervention and ensure continuity of care (229). Experts from various disciplines jointly assess patients to ensure that exercise intervention plans are both scientifically sound and safe. Through comprehensive geriatric assessment, the team can understand a patient’s physical function, nutritional status, and level of social support, allowing for the selection of appropriate exercise types and intensities. Multidisciplinary collaboration also ensures that exercise interventions are integrated throughout the entire cancer treatment process. Preoperative Prehabilitation (Before Treatment): Exercise interventions implemented at this stage can significantly enhance physiological reserves prior to surgical stress (230). Postoperative and Chemotherapy-stage Rehabilitation (During/After Treatment): Tailored exercises help patients recover more quickly, manage treatment-related toxicities, and restore functional capacity (231). During long-term follow-up, maintaining exercise habits helps prevent cancer recurrence and age-related frailty (232). This continuity maximizes the benefits of exercise interventions and reduces the likelihood of treatment interruptions or missed opportunities. In practice, effective collaboration mechanisms and standardized procedures are required for successful multidisciplinary cooperation. Hospitals can establish geriatric oncology exercise rehabilitation units or hold regular multidisciplinary team meetings to discuss eligible cases and develop exercise plans. At the same time, unified exercise intervention guidelines and operational protocols should be developed to define each discipline’s responsibilities and ensure seamless handover between professionals. Healthcare providers should also receive training on the risks and benefits of exercise to understand the value of these interventions and gradually integrate exercise prescriptions into standard oncology treatment pathways. Through the establishment of a systematic management framework, exercise interventions can continuously promote the comprehensive and precise care of older cancer patients.

## 6 Summary and future outlook

Current evidence suggests that exercise may beneficially influence several aging-related physiological and microenvironmental factors relevant to cancer, including metabolism, inflammation, immunity, skeletal muscle, and treatment tolerance. Although exercise medicine is well established as a supportive component of postoperative rehabilitation, its potential as a comprehensive intervention spanning cancer prevention, treatment, and recovery remains at an early stage. As the field evolves, it may help reshape cancer-care systems by complementing tumor-directed treatment with strategies that strengthen host physiological resilience. Despite the promising potential of exercise in the treatment of cancer in older adults, several scientific challenges remain to be explored. First, the molecular mechanisms by which exercise regulates the interaction between aging and tumor development have not yet been fully elucidated. Investigating key signaling pathways and targets through multi-omics approaches may provide a theoretical foundation for developing new therapies that simulate the beneficial effects of exercise (233, 243). To resolve existing knowledge gaps, future research should leverage high-resolution spatial transcriptomics to visually map the precise mechanical and exerkine gradients across the tumor architecture, alongside utilizing single-cell RNA sequencing (scRNA-seq) to uncover the exact cellular networks and transcriptomic shifts of shifting immune phenotypes inside the aged TME in response to regular physical training. Second, the patterns of synergy between exercise and traditional cancer treatments are not yet clearly defined. For various cancer types, the optimal timing, intensity, duration of exercise interventions, and how they integrate with chemotherapy or immunotherapy still require further study. Real-world evidence is also essential, particularly for evaluating the long-term outcomes and feasibility of exercise interventions across diverse populations. Such studies should assess the impact of exercise adherence on survival and functional outcomes, offering valuable data to support clinical decision-making. Furthermore, bridging the gap between research protocols and daily patient care will increasingly rely on wearable technologies. As discussed regarding digital monitoring, these devices can continuously track the real-world ‘dose-response’ of physical activity, providing objective data to dynamically adjust personalized exercise prescriptions. Finally, interdisciplinary collaboration is especially important. Integrating knowledge from geriatrics, oncology, exercise physiology, and related fields, and leveraging digital technology and artificial intelligence to develop personalized exercise prescriptions, will further promote the application of exercise in the field of precision oncology (234, 235).

Looking ahead, it is imperative to consider how to translate the findings of exercise intervention research into practical medical applications and fully integrate them into the routine care of older adults with cancer. Advancing exercise interventions through clinical pathways and health policy frameworks is essential to achieving this goal. This process requires the support of healthcare administrators and policymakers, including the incorporation of recommendations for appropriate physical activity for older patients into oncology clinical guidelines, the development of rehabilitation protocols specifically tailored to geriatric oncology patients, and the exploration of including exercise rehabilitation services within medical insurance and public health systems to alleviate the financial burden on patients. Policy support can facilitate the adoption of exercise as a routine component of cancer care and enhance awareness among clinicians and patients regarding the therapeutic value of physical activity. Establishing a standardized, monitorable, and evaluable exercise intervention framework is critical to ensure consistent and high-quality implementation across various regions and healthcare institutions. Based on current scientific evidence, exercise prescriptions should be developed for different clinical phases, including the perioperative period, chemotherapy, and remission, with clearly defined parameters for safety monitoring. Simplified assessment tools and prescription templates should assist frontline medical staff in designing individualized exercise plans for older patients. Digital health technologies can be leveraged to create a unified data reporting and feedback system, integrating exercise information into electronic medical records to support continuous monitoring, quality control, and strategy refinement. In addition to hospital-based strategies, community and family resources should be mobilized to build a three-tiered support model encompassing hospitals, community health services, and households, ensuring that patients receive continuous exercise intervention and support. Hospital resources alone are insufficient for older patients with limited mobility or those requiring long-term rehabilitation. After the initial assessment and plan development, hospitals should collaborate with community health centers and fitness facilities to provide accessible and affordable exercise environments and professional guidance. Trained community rehabilitation personnel should supervise patient exercise based on hospital-issued plans and report progress regularly. At the household level, family-based rehabilitation training should be implemented to enhance caregivers’ awareness of the importance of physical activity, enabling them to serve as advocates and facilitators of patient adherence, thereby ensuring continuity of exercise support beyond the hospital setting.

In summary, the development of a comprehensive geriatric oncology exercise medicine system requires coordinated advancement in policy formulation, clinical guidelines, and resource allocation. Once exercise interventions are established as routine practice, they will become an indispensable component of comprehensive cancer care, much like perioperative nutritional support and palliative care. Looking ahead, with the ongoing trends of population aging and shifts in cancer epidemiology, exercise medicine will become increasingly integrated with oncology, enabling a patient-centered, holistic approach to treatment and supporting older cancer patients in their pursuit of recovery and longevity.
